# Postoperative Seizure in Patients with Malignant Glioma Undergoing Tumor Resection with Intraoperative Mapping: Risk Factors, Management Strategies, and the Utility of Bayesian Analysis in a Case-Control Cohort

**DOI:** 10.21203/rs.3.rs-8705953/v1

**Published:** 2026-02-10

**Authors:** Yifei Sun, Mina Lobbous, Kristen Riley, Pedram Maleknia, John Stein, Nicholas Laskay, Travis Atchley, James Markert, Dagoberto Estevez-Ordonez, Burt Nabors

**Affiliations:** University of Alabama at Birmingham; Cleveland Clinic; University of Alabama at Birmingham; Baylor College of Medicine; University of Cincinnati Medical Center; Columbia University Irving Medical Center; Emory University Hospital; University of Alabama at Birmingham; University of Miami Health System; University of Alabama at Birmingham

**Keywords:** glioma, resection, seizure, risk factors, management, bayesian analysis, intraoperative mapping

## Abstract

**BACKGROUND:**

Postoperative seizures can occur secondary to cortical irritation from malignant glioma resection or from direct electrical stimulation of the cortical surface during intraoperative brain mapping. A paucity of literature exists with regards to the use of appropriate seizure risk-reduction strategies for this patient population. The objective of the study was to identify primary risk factors for early and late postoperative seizures following intraoperative brain mapping.

**METHODS:**

The authors performed a case-control study with 30 patients who had postoperative clinical seizures within 6 months following craniotomies with intraoperative mapping for glioma resection from 2013 to 2021 at a single academic institution. An unmatched control population of all patients (n=52) who had undergone craniotomies with ICM during the same period and had no clinical seizures within 6 months following their operation were used for comparative analysis. Primary endpoint was any postoperative seizure within 6 months of surgery. Outcomes were analyzed both via frequentist and Bayesian statistical approaches.

**RESULTS:**

Bayesian analysis using non-informative priors demonstrated that the probability of an odds ratio (OR) > 1 for prior history of seizures being a risk factor for postoperative seizures is 73%. The probability that OR < 1 for a patient with post op seizures who underwent motor mapping was 91%. If patients experienced an intraoperative seizure during mapping, the probability of having a postoperative seizure was 84%. Probability that awake mapping is protective of post op seizure when compared to asleep mapping is 88%. Complex anti-epileptic drug (AED) regimen (increasing dose + adding additional AEDs or 2 dose adjustments) had 64% probability of protection from late postoperative seizures.

**CONCLUSION:**

Patients with a preoperative history of seizures may be at higher risk for postoperative seizures. More aggressive perioperative seizure prophylaxis may provide a protective benefit from postoperative seizures in patients who undergo intraoperative mapping.

## INTRODUCTION

Gliomas are the leading cause of malignant brain tumors and remain a challenging and heterogeneous tumor population to treat. Currently, maximal resection of these tumors followed by adjuvant therapies such as radiotherapy and chemotherapy remain the mainstay of treatment. Preservation of neurological function with this approach is essential and is associated with improved outcomes.^1,2^ To achieve this surgical goal, intraoperative cortical mapping (ICM) is frequently performed in order to maximize tumor resection while protecting eloquent, non-resectable regions of tumor.^3^

However, intraoperative and postoperative seizures remain unacceptably common adverse events in ICM despite improvements in technique.^4,5^ These seizures occur in 17% to 50% of patients undergoing resection with ICM, with seizures seen more often with tumors affecting the frontal, parietal, and temporal lobes on the cortical surface.^6–10^ There is a paucity of understanding of risk and protective factors for seizure occurrence following glioma resection with ICM. Recent studies have shown that longer stimulus durations can induce seizures.^11,12^ Studies have found that patient factors such as preoperative epilepsy may also predispose to seizures intraoperatively, and there is concern that intraoperative seizures may predispose to further postoperative events, though few studies have specifically investigated this relationship.^5,11–13^ Preoperative seizure history has also been found to increase the risk of postoperative seizures following ICM as well.^14^ While some studies suggest that appropriate preoperative seizure prophylaxis may decrease the harmful sequelae of postoperative seizures, there also exists a paucity of data on the appropriate management strategy for seizure prevention specifically in patients undergoing ICM.^6,15–18^

The primary aims of this study were to identify risk and protective factors for postoperative seizures in patients undergoing ICM via awake or asleep craniotomies and to provide insight into appropriate preventive management strategies. We hypothesized that a postoperative anti-epileptic drug (AED) regimen (either addition of new AEDs or dose increase) would be protective against postoperative seizures in this patient population. We also highlight the potential utility of a Bayesian analysis approach through a post-hoc comparison with our pre-hoc frequentist analysis results.

## METHODS

This retrospective study was designed as a case-control study with approval from the Institutional Review Board at our institution, and informed consent was waived given the nature of the study. This manuscript was prepared in accordance to the EQUATOR network and The Strengthening the Reporting of Observational Studies in Epidemiology (STROBE) reporting guidelines.^19,20^

### Variables and Data Management

We identified 30 patients who had postoperative clinical seizures within 6 months following craniotomies with ICM for supratentorial brain tumor resection between 2013 to 2021 performed by 3 neurosurgeons at our institution. We used an unmatched control population of all patients (n = 52) who had undergone craniotomies with ICM during the same period and who had no clinical seizures within 6 months following their operation.

Relevant variables were defined via consensus expertise by senior authors (M.L., K.R., B.N., and J.M.M.) and literature review on known postoperative seizure risk factors in this patient population. All variables were collected via electronic chart review. Most variables were binary or categorical except for age and income, all groups were defined according to standard practices to facilitate comparison. AED regimen categories were defined preoperatively (categorized as either monotherapy or polytherapy (at least 2 different medications) AEDs), and postoperative AED regimen (defined as any adjustment to AEDs or lack thereof at time of discharge). AED regimens were further categorized as monotherapy AED regimen, which was defined as only one medication, polytherapy AED regimen, which was defined as at least 2 medications, and complex AED regimen, which was defined as either increasing dose and adding additional AEDs or at least 2 dose adjustments postoperatively. Other variables collected included patient demographics, patient clinical and tumor characteristics, relevant dates, anesthesia modality (awake vs. asleep), mapping type and time, extent of resection, prior history of seizure and characteristics, intraoperative seizures, and AED regimen. We performed multiple imputations (incorporating previous outcome values and relevant demographic and clinical variables) and last observation carried forward on the outcome variable for 7 patients lost to follow up at 6 months with similar results.^21,22^ Further details of variable collection and data management can be found in the Supplementary Digital Content.

The primary outcome of interest was any clinical postoperative seizure within a 6-month period following surgical resection with ICM. Patients were assessed for postoperative seizures immediately after surgery and again at the 1- and 6-month outpatient follow-up visits. Seizures were defined as either focal or generalized seizures identified in the chart by observation, clinical and history evaluation by a neurologist, or electroencephalography (EEG). Early seizures were defined as seizures occurring within 7 days post operatively and late seizures were defined as seizures occurring after 7 days within 6 months post operatively.

### Statistical Methods

This study with 82 participants had 0.8 power to detect an odd ratio (OR) of 0.28 and 6.4 assuming the proportion of controls exposed was 70% and OR of 0.16 and 3.6 assuming the proportion of controls exposed was 30%. Descriptive statistics were reported as numbers and percentages for categorical variables Age, given its bimodal distribution, was reported as means and interquartile ranges. Univariable comparisons were performed using Wilcoxon rank-sum test for age and Pearson’s χ2 or Fisher’s exact test when appropriate. Multivariable analysis was done using logistic regression for binary outcome with variables selected by consensus based on either known association or suspected potential clinical association with the outcome. To account for unmeasured or uncontrolled confounding, sensitivity analysis was performed using the methodology proposed by VanderWeele and Ding (Supplementary Digital Content, Figures S1-S2).^23^

### Post-hoc Bayesian Analysis

To aid in the identification of protective and risk factors, we conducted a post hoc Bayesian analysis. We adhered to the when to Worry and how to Avoid the Misuse of Bayesian Statistics (WAMBS-v2) guidelines.^24^ A minimally informative reference prior (assumed mean coefficient 0 – OR 1 – and variance 10,000) was used to produce results dependent on data from this study alone. This noninformative prior regards all possible OR values to be equally likely and is equivalent to holding no prior belief on whether variables selected for analysis are either protective or risk factors for the specified outcome. An informative data-derived prior (mean coefficient 0.732 – OR 2.08 – for a history of seizures as a risk factor for postoperative seizures in a multivariable model and assumed variance of 1) with data from Eseonu *et al*. (2017)^14^ was also used. Separate Bayesian models were run for each prior distribution. Each model used the same logistic regression construct (with tested assumptions) as in the primary analysis. Markov chain Monte Carlo (MCMC) with random-walk Metropolis-Hasting sampling algorithm (with 4 chains, 25,000 iterations burn-in and 40,000 total saved iterations per chain by saving every 15i + 1 iteration) was used to derive OR estimates and highest posterior density (HDP) 95% credible intervals (CrIs) from the median and to estimate the posterior probabilities of exposure variables exceeding thresholds for protective or risk factors as appropriate.^24,25^ Model convergence was assessed visually (using trace, histogram, density, and autocorrelation plots) and using the Gelman-Rubin statistic (*Rc-hat*) *Rc-hat* values, plots, and effective sample size for all parameters as well as results from sensitivity analysis performed can be found in the Supplemental Digital Content (Supplementary Digital Content, Tables S5-S11, Figure S3).^26,27^

All statistical analyses were done using STATA v17.^28^ Further details of this analysis can be found in the Supplementary Digital Content.^24–27^

## RESULTS

### Patient Characteristics

We identified 82 patients from January 2013 to March 2021 with supratentorial gliomas located in eloquent areas of the brain who had undergone craniotomy for resection with ICM. Thirty (37%) of these patients had at least one postoperative seizure within 6 months of initial resection (Table 1).

Demographics of the patient and characteristics of the tumors themselves were very similar (Table 1). The mean age was 51.0 (IQR 35.4–60.5) years in the control group and 54.4 (38.7–67.7) years amongst the seizure group (p = .26). Amongst the controls, 26 (50%) had a preoperative history of seizures (of which 73% were on preoperative AEDs) compared to 20 (67%) of the seizure cases (87% on preoperative AEDs) (p= .14). Six (12%) of the controls and 4 (13%) of the seizure group had intraoperative seizures (p=.81). Most of the patients in both groups were placed on levetiracetam postoperatively [50 (96%) v. 28 (93%)]. There were more patients amongst the seizure cases who were placed on lacosamide postoperatively [6 (12%) vs 17 (57%), p< .001]. Other patient characteristics, pathological diagnosis, intraoperative mapping/resection techniques were found to be similar between groups (Table 1).

**Figure 1:**
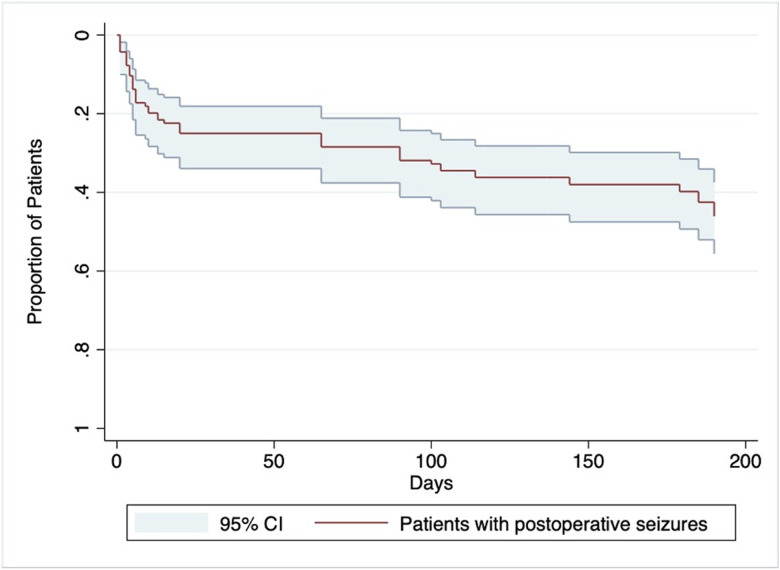
Kaplan-Meier estimate of the proportion of patients who experienced at least one postoperative clinical seizure within 6 months

A Kaplan-Meier estimate of the proportion of patients who experienced at least one postoperative clinical seizure within 6 months was generated. The median time to first postoperative seizure was 7.5 days, with 50% of seizures occurring early. A range of 0 to 190 days to seizure recurrence was observed. Incidence rate of postoperative seizures was 2.61 seizures per 1,000 person-days (Fig. 1).

### Primary Outcome

**Figure 2:**
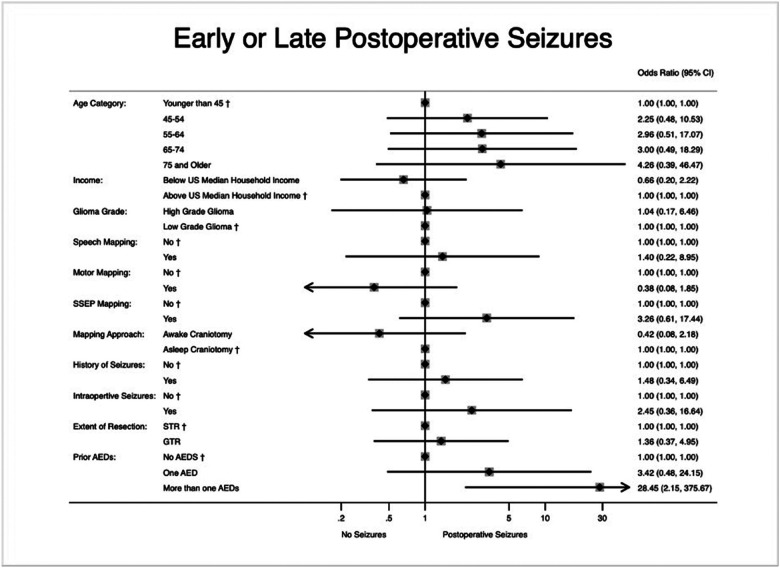
Multivariate logistic regression for factors associated with postoperative seizure

Results of sensitivity analysis using methodology proposed by VanderWeele and Ding are presented in the Supplementary Digital Content.^23^ After multivariate logistic regression analysis, we found that patients with a preoperative history of seizures and concurrently taking more than one AED were more likely to have postoperative seizures following surgery (OR 28.45, 95% CI 2.15–375.57, p = 0.011). However, history of preoperative seizures alone was not an independent risk factor for postoperative seizures (OR 1.48, 95% CI 0.34–6.49, p = 0.606). Figure 2 shows the multivariate logistic regression comparison between cases and controls for postoperative seizure (Supplementary Digital Content, Table S2).

### Post-hoc Analysis of Secondary Outcomes

**Figure 3:**
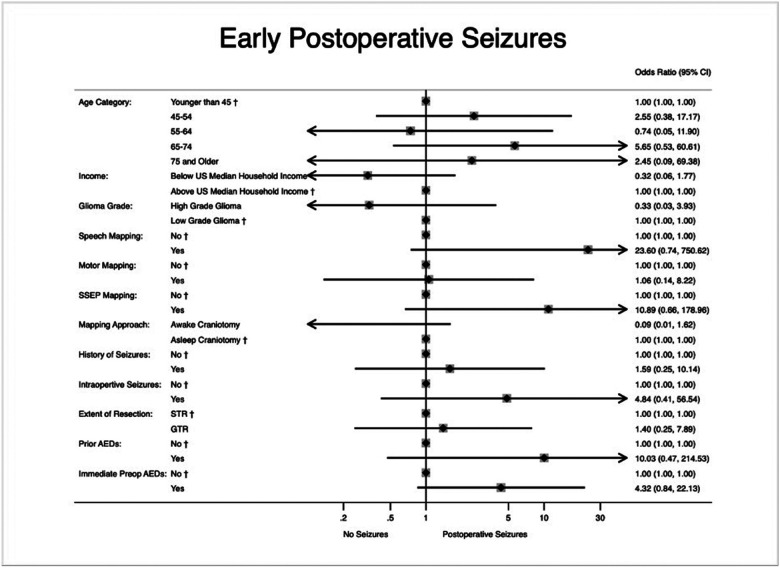
Multivariate logistic regression for factors associated with early postoperative seizure

Post-hoc analysis was performed for patients with early seizures and patients with late seizures (Supplementary Digital Content, Tables S3-S4). Upon multivariate logistic regression analysis, preoperative history of seizures, history of preoperative AED usage, and seizures intraoperatively during the mapping portion of the procedure did not confer a higher odds of having early postoperative seizures. Results of this analysis are shown in Fig. 3.

**Figure 4:**
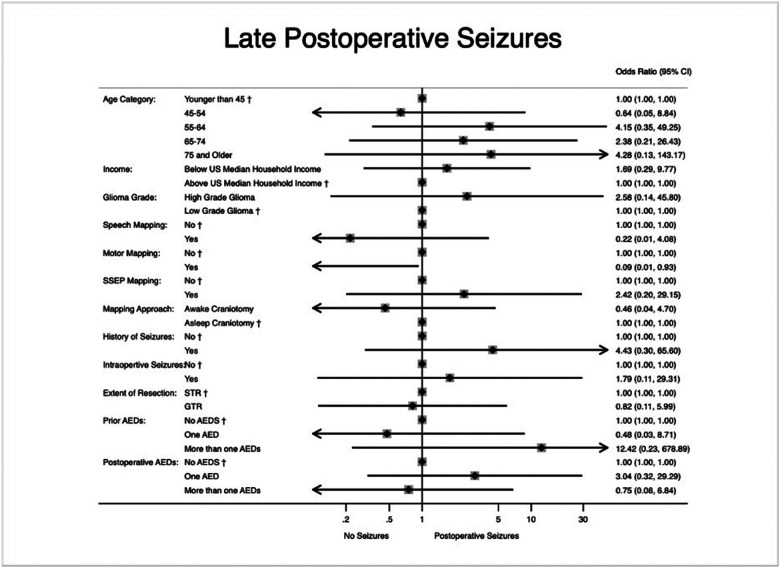
Multivariate logistic regression for factors associated with late postoperative seizures

On multivariate logistic regression analysis in patients with late postoperative seizures, patients were found to have a lower odds ratio of having motor mapping during their resection (OR 0.08, 95% CI 0.01–0.86, p=.037). This association was not seen with regards to speech mapping (OR 0.26, 95% CI 0.01–6.33, p=.409). Results of this analysis with odds ratios and 95% confidence intervals can be seen in Fig. 4.

### Post-hoc Bayesian Analysis without Informative Priors

ORs with 95% credible intervals are shown in Table 2. Several hypotheses were tested. The probability of a prior history of seizures being a risk factor (OR > 1) for postoperative seizures was 72.5%. The probability that the use of motor mapping was protective (OR < 1) was 91.5%. The probability that awake mapping is protective of postoperative seizures when compared to asleep mapping is 88.1%. The probability that gross total resection was a risk factor for postoperative seizures when compared to those who had subtotal resections was 70.6%. The probability of intraoperative seizures being a risk factor for postoperative seizures was 84.4%. Interestingly, the probability that sensory or language mapping was a risk factor for postoperative seizures was 94.2% and 65.8%, respectively. Monotherapy AED with dose increase alone had a 18.87% probability of acting as a protective factor for late postoperative seizures. A complex AED regimen had 63.35% probability of acting as a protective factor from late postoperative seizures.

### Post Hoc Bayesian Analysis using Informative Priors

ORs with 95% credible intervals are shown in Table 2. Eseonu *et al*. (2017)^14^ reports a history of seizures as an independent risk factor for postoperative seizures, and we subsequently updated our priors with this information. The probability that OR > 1 for prior history of seizures being a risk factor for postoperative seizures was updated to 82.0%, from 72.5% using non-informative priors (Table 2).

## DISCUSSION

### Risk Factors

In traditional frequentist analysis, we found that preoperative polytherapy AED regimen for preoperative seizure control was a risk factor for postoperative seizures after glioma resection with ICM, but the presence of preoperative seizures alone was not an independent risk factor (Fig. 2). This may be that patients requiring multiple AEDs have poorly controlled or refractory seizures preoperatively and surgery only serves to exacerbate the condition.^29,30^

Upon Bayesian analysis without informative priors, we observed that a history of prior seizures, intraoperative seizures, sensory mapping, language mapping, gross total resection rather than subtotal resection, being of older age, and having a history of monotherapy AED or polytherapy AED were implicated as potential risk factors for seizure following glioma resection with ICM. Several of these results are in line with findings by Conte *et al*. (2015)^31^, who found frontal tumor location, antiepileptic polytherapy, intraoperative seizures during mapping, and postoperative blood products on imaging to be independent predictors of immediate postoperative seizures. Of note, a history of prior seizures was a potential risk factor, likely because a history of prior seizures may indicate some underlying seizure pathology that would only be aggravated by ICM during glioma resection.^32^ Gross total resection rather than subtotal resection has an increased likelihood of being a risk factor possibly due to resection closer to epileptogenic regions, increasing the likelihood of potential irritation to these regions. Intraoperative seizures were found to have increased likelihood of being a risk factor possibly for a combination of reasons. High-intensity currents applied directly on the cortex can result in after-discharges (ADs), which can then propagate into clinical seizures, and may occur due to pathological network connectivity, among others.^33–37^ Thus, this seizure provocation that triggered the intraoperative seizure likely persisted into the post-operative period, predisposing the patient to further seizures.

Sensory mapping and language mapping were also found to be potential risk factors. These findings can be explained by a study by Chaichana et. al (2009)^38^, who found that parietal lobe infiltration by glioma predicted poor seizure control; thus, sensory mapping implies a tumor involvement in a region involved with poor seizure outcomes. Language mapping centers are found in regions of the brain highly associated with seizures as well, explaining the high probability of being a seizure risk factor.^39^

Interestingly, we further found awake mapping to be a potential protective factor for seizures after glioma resection with ICM. This observation can be explained by a potentially increased resolution of eloquent areas and problematic foci during awake mapping rather than asleep.^40^ We also found motor mapping to be a likely protective factor. This may be because motor mapping may be a surrogate for tumor location, as these tumors may have a significant frontal component. In such circumstances, a greater extent of resection may be achievable especially for tumors isolated to the frontal lobes. This more aggressive resection may decrease the odds of seizures in the long-term once patients are past the perioperative period.^41^

However, these findings may emphasize a couple of potential confounders issues. The definition of seizures is heterogeneous and often subjective; therefore, there may be a misclassification bias. If patients have tumors extending into clinically silent or non-eloquent regions, then their incidence of seizures may be under-reported or clinically silent altogether, affecting our data.

### Management of Seizures following Glioma Resection with ICM

Prophylaxis against intra- and post-operative seizures remains a contested issue.^9,42,43^ Dineen *et al*. (2019)^13^ found that preoperative loading with intravenous AEDs (levetiracetam, fosphenytoin, lacosamide) decreased the odds of an intraoperative seizure (OR 0.55, p = 0.009). However, a prospective randomized trial by Wu *et al*. (2013)^15^ found that postoperative prophylaxis with phenytoin did not decrease the risk of seizures and was associated with increased medication adverse events. Al-Dorzi *et al*. (2017)^9^ similarly did not find that postoperative AED prophylaxis decreased seizure incidence.

We had hypothesized that the initiation of or dose increase in a postoperative AED regimen would be protective against seizures in patients who had resection with ICM. However, via the frequentist approach, we did not find a significant association. But via a Bayesian model, the relationship can be more clearly delineated. We found that AED monotherapy with a postoperative dose increase had less than a 20% probability of protecting against seizures. However, a complex AED regimen (increasing dose + adding additional AEDs or 2 dose adjustments postop) had 63.35% probability of protecting against late postoperative seizures. This discrepancy between frequentist and Bayesian approaches emphasizes one of the other aims of this study.

### The Utility of Bayesian Analysis

Bayesian analysis re-allocates relative credibilities within the set of considered possibilities.^44^ There are several advantages to using Bayesian analysis such as allowing researchers to incorporate prior information into current analysis and providing a conceptually simpler multilevel analysis.^44–46^ However, to conduct Bayesian statistics, prespecified “priors” is often required. In the present study, we performed analysis both with and without information priors (Table 2), and we relied on various thresholds risk factor thresholds and found the probability of preoperative seizure history increasing the odds of postoperative seizures (OR > 1) to be 72.5%. After accounting for findings reported by Eseonu *et al*. (2017)^14^ the probability of preoperative seizures increasing the odds of postoperative events rose to 82.0%.^14^ Bayesian methods allow for the probabilities of associations to be reported and adjusted given prior information (Fig. 3). This framework may be useful in the study of topics that may be otherwise underpowered and may be clinically useful when utilizing different thresholds in order to account for clinical experience. Although a frequentist analytic framework is far more common in the neurosurgical literature, this study highlights the potential utility of using a Bayesian framework. Results from this approach are more intuitive in a clinical setting, especially when considering extant associations or strong *a priori* clinical assumptions, and can be very useful in settings with few participants often at risk of an underpowered analysis

### Limitations

This is a single-institution, retrospective study with all the limitations therein. Multiple oncologic neurosurgeons were included in this series, and differences in intraoperative technique or aggressiveness in ICM may be reflected in the data. However, given that we did not find a significant association between ICM and postoperative seizures, we suspect these technical differences to be marginal. The definition of seizure used as the primary outcome of this study included both subjective and objective criteria. As such, the frequency of these events may be under- or over-reported. However, similar limitations are noted throughout literature and the use of solely objective criteria (EEG findings correlating with clinical events) would likely greatly underestimate seizure frequency. For the present study, we chose to potentially overestimate clinical seizures. For our Bayesian analysis, there was a paucity of informative priors. As such, the majority of the analyses assume non-informative priors and may under-value the results of this technique.

## CONCLUSION

Patients with a preoperative history of seizures may be at higher risk for postoperative seizures, and aggressive perioperative seizure prophylaxis may provide a protective benefit from postoperative seizures in patients who undergo intraoperative mapping.

## Supplementary Material

This is a list of supplementary files associated with this preprint. Click to download.

- revisedSupplementalDigitalContent.docx

## Figures and Tables

**Table 1:** Patient Characteristics

Variable		No Seizure	Seizure	p-value
		n = 52	n = 30	
Gender, N (%)	Female	26(50%)	11(37%)	0.24
	Male	26(50%)	19(63%)	
Age, Mean (IQR)		51.1(35.4–60.5)	54.4(38.7–67.7)	0.26
Age Categories	< 45	24(46%)	9(30%)	0.66
	45–54	9(17%)	7(23%)	
	55–64	9(17%)	6(20%)	
	65–74	7(13%)	5(17%)	
	75+	3(6%)	3(10%)	
Race	White	42(81%)	25(83%)	0.65
	African American	8(15%)	3(10%)	
	Other	2(4%)	2(7%)	
Income	Below U.S. Median Household Income	34(65%)	17(57%)	0.43
	Above U.S. Median Household Income	18(35%)	13(43%)	
RUCA	Metropolitan	38(73%)	21(70%)	0.81
	Micropolitan	6(12%)	5(17%)	
	Small Town/Rural	6(12%)	4(13%)	
Glioma Type	High Grade Glioma	41(79%)	26(87%)	0.38
	Low Grade Glioma	11(21%)	4(13%)	
Diagnosis	GBM	35(67%)	21(70%)	0.86
	Anaplastic Oligodendroglioma	2(4%)	2(7%)	
	Diffuse Astrocytoma	5(10%)	2(7%)	
	Oligodendroglioma	3(6%)	2(7%)	
	Anaplastic Astrocytoma	3(6%)	3(10%)	
	LGG, NOS	3(6%)	0(0%)	
	Anaplastic Ganglioglioma	1(2%)	0(0%)	
Lesion Laterality	Left Side	35(67%)	19(63%)	0.71
	Right Side	17(33%)	11(37%)	
Disease Progression		7(13%)	5(17%)	0.69
History of Disease Recurrence		18(35%)	7(23%)	0.29
History of Seizures Pre-op		26(50%)	20(67%)	0.14
Type of Seizure Pre-op	None	26(50%)	10(33%)	0.01
	Focal	11(21%)	16(53%)	
	Generalized	15(29%)	4(13%)	
Pre-op AED (PPXvsTRX)	No AED Prior to Surgery	14(27%)	4(13%)	0.27
	Seizure Prophylaxis	12(23%)	6(20%)	
	Seizure Treatment	26(50%)	20(67%)	
Pre-op AED Regimen	No AEDs	14(27%)	4(13%)	
	One AED	34(65%)	18(60%)	
	Two or More AEDs	4(8%)	8(27%)	0.041
Anesthesia Type	Awake	42(81%)	22(73%)	0.43
	Asleep	10(19%)	8(27%)	
Mapping	Motor	40(77%)	22(73%)	0.6
	Speech	27(52%)	15(50%)	0.8
	SSEP	10(19%)	9(30%)	0.29
Mapping Time	30 minutes or less	23(44%)	16(53%)	0.16
	Greater than 30 minutes	19(37%)	6(20%)	
Immediate Pre-Monitoring	AED Adjustment/Increase	14(27%)	10(33%)	0.54
Extent of Resection	STR	32(62%)	18(60%)	1
	GTR	20(38%)	12(40%)	
Neurosurgeon	Neurosurgeon No. 1	39(75%)	25(83%)	0.73
	Neurosurgeon No. 2	12(23%)	5(17%)	
	Neurosurgeon No. 3	1(2%)	0(0%)	
Seizures - Intraoperative		6(12%)	4(13%)	0.81
Seizures - Immediately post-op		0(0%)	15(50%)	< 0.001
AED at Discharge - Adjustment		34(65%)	26(87%)	0.036
AED at Discharge - Adjustment	No change in pre-op AED regimen or dose	18(35%)	4(13%)	< 0.001
	Monotherapy with dose increase	24(46%)	7(23%)	
	Complex Adjustment	10(19%)	19(63%)	
AED Type	Levetiracetam	50(96%)	28(93%)	0.57
	Lacosamide	6(12%)	17(57%)	< 0.001
	Other AED	6(12%)	14(47%)	< 0.001

**Table 2:** Probability of Odds Ratio Estimated by Bayesian Analysis in Patients Undergoing Intraoperative Mapping

Risk Factors						
		Posterior Probability That True OR Is >Specified Threshold, %				
	Posterior Median OR (95% Credible Interval)	OR > 1	OR > 1.2	OR > 1.5	OR > 1.8	OR > 2
**Minimally Informative Priors**						
Prior History of Seizures	2.42 (0.11–6.89)	72.5	64.7	54.3	45.6	40.7
High Grade Glioma	1.95 (0.02–6.37)	52.7	45.8	37.7	31.4	27.9
Age 55–64	6.67 (0.09–21.25)	91.5	88.2	83.2	78.3	75.0
Age 65–74	6.82 (0.10–22.03)	90.7	87.3	82.1	77.0	73.7
Age > 75	17.59 (0.01–63.88)	90.6	88.2	84.7	81.5	79.3
Language Mapping	2.86 (0.02–9.39)	65.8	59.2	50.8	43.9	40.1
Sensory Mapping	7.47 (0.15–23.42)	94.2	91.6	87.1	82.5	79.5
Intraoperative Seizures	5.93 (0.04–19.83)	84.4	80.2	74.1	68.4	64.9
Gross Total Resection	1.98 (0.14–5.15)	70.6	61.6	49.9	40.1	34.8
History of AED monotherapy	9.98 (0.10–34.01)	92.5	89.7	85.4	81.2	78.4
History of taking 2 or more AEDs	275.33 (0.22–1007.972)	99.9	99.8	99.7	99.6	99.5
**Data Derived Priors**						
Prior History of Seizures		82.0	73.6	61.1	50.1	43.6
Protective Factors						
		Posterior Probability That True OR Is <Specified Threshold, %				
	Posterior Median RR (95% Credible Interval)	OR < 1	OR < 0.8	OR < 0.6	OR < 0.5	OR < 0.4
**Minimally Informative Priors**						
Motor Mapping	0.44 (0.01–1.28)	91.5	86.9	79.0	72.7	63.8
Awake Mapping	0.51 (0.01–1.56)	88.1	82.7	74.0	67.4	58.4

## Data Availability

Data is available upon reasonable request.

## Abbreviations

AED: Anti-epileptic drug
AD: After-Discharges
ICM: intraoperative cortical mapping

## References

[1] Hervey-Jumper SL, Berger MS (2016) Maximizing safe resection of low- and high-grade glioma. Journal of Neuro-Oncology. /11 2016;130(2):269–282. 10.1007/s11060-016-2110-4 27174197

[2] Ma R, Taphoorn MJB, Plaha P (2021) Advances in the management of glioblastoma. J Neurol Neurosurg Psychiatry 10(10):1103–1111. 10.1136/jnnp-2020-325334. /01 2021 34162730

[3] Wang Y-C, Lee C-C, Takami H (2019) Awake craniotomies for epileptic gliomas: intraoperative and postoperative seizure control and prognostic factors. J Neurooncol 2019/05(3):577–586. 10.1007/s11060-019-03131-0 30805752

[4] Szelényi A, Bello L, Duffau H Intraoperative electrical stimulation in awake craniotomy: methodological aspects of current practice. NeuroSurg Focus. 2010/02/01 2010;28(2):E7. 10.3171/2009.12.FOCUS09237 20121442

[5] Nossek E, Matot I, Shahar T (2013) Intraoperative seizures during awake craniotomy: incidence and consequences: analysis of 477 patients. Neurosurgery. /07 2013;73(1):135–140; discussion 140. 10.1227/01.neu.0000429847.91707.97 23615101

[6] Eseonu CI, Eguia F, Garcia O, Kaplan PW, Quiñones-Hinojosa A (2018) Comparative analysis of monotherapy versus duotherapy antiseizure drug management for postoperative seizure control in patients undergoing an awake craniotomy. J Neurosurg Jun 128(6):1661–1667. 10.3171/2017.1.Jns162913 28621631

[7] Chaichana KL, Pendleton C, Zaidi H (2013) Seizure control for patients undergoing meningioma surgery. World Neurosurgery. /04 2013;79(3–4):515–524. 10.1016/j.wneu.2012.02.051 22469524

[8] Tanriverdi T, Kemerdere R, Baran O Long-term surgical and seizure outcomes of frontal low-grade gliomas. Int J Surg. 2016/09 2016;33 Pt A:60–64. 10.1016/j.ijsu.2016.07.065 27475744

[9] Al-Dorzi HM, Alruwaita AA, Marae BO (2017) Incidence, risk factors and outcomes of seizures occurring after craniotomy for primary brain tumor resection. Neurosciences (Riyadh) 2017/04(2):107–113. 10.17712/nsj.2017.2.20160570 28416781 PMC5726815

[10] Lynam LM, Lyons MK, Drazkowski JF (2007) Frequency of seizures in patients with newly diagnosed brain tumors: a retrospective review. Clinical Neurology and Neurosurgery. /09 2007;109(7):634–638. 10.1016/j.clineuro.2007.05.017 17601658

[11] Sartorius CJ, Berger MS (1998) Rapid termination of intraoperative stimulation-evoked seizures with application of cold Ringer’s lactate to the cortex. Technical note. J Neurosurg. /02 1998;88(2):349–351. 10.3171/jns.1998.88.2.0349 9452250

[12] Szelényi A, Joksimovic B, Seifert V (2007) Intraoperative risk of seizures associated with transient direct cortical stimulation in patients with symptomatic epilepsy. J Clin Neurophysiology: Official Publication Am Electroencephalographic Soc 2007/02(1):39–43. 10.1097/01.wnp.0000237073.70314.f7 17277576

[13] Dineen J, Maus DC, Muzyka I (2019) Factors that modify the risk of intraoperative seizures triggered by electrical stimulation during supratentorial functional mapping. Clin Neurophysiol. /06 2019;130(6):1058–1065. 10.1016/j.clinph.2019.03.006 30930194

[14] Eseonu CI, ReFaey K, Garcia O, John A, Quiñones-Hinojosa A, Tripathi P (2017) Awake Craniotomy Anesthesia: A Comparison of the Monitored Anesthesia Care and Asleep-Awake-Asleep Techniques. World Neurosurg 2017/08:104:679–686. 10.1016/j.wneu.2017.05.053 28532922

[15] Wu AS, Trinh VT, Suki D (2013) A prospective randomized trial of perioperative seizure prophylaxis in patients with intraparenchymal brain tumors. J Neurosurg. /04 2013;118(4):873–883. 10.3171/2012.12.JNS111970 23394340 PMC4083773

[16] Klimek M, Dammers R (2010) Antiepileptic drug therapy in the perioperative course of neurosurgical patients. Curr Opin Anaesthesiol. /10 2010;23(5):564–567. 10.1097/ACO.0b013e32833e14f2 20689411

[17] Siomin V, Angelov L, Li L, Vogelbaum MA (2005) Results of a survey of neurosurgical practice patterns regarding the prophylactic use of anti-epilepsy drugs in patients with brain tumors. J Neurooncol 2005/09(2):211–215. 10.1007/s11060-004-6912-4 16193395

[18] Rossetti AO, Stupp R (2010) Epilepsy in brain tumor patients. Current Opinion in Neurology. /12 2010;23(6):603–609. 10.1097/WCO.0b013e32833e996c 20733482

[19] von Elm E, Altman DG, Egger M (2007) The Strengthening the Reporting of Observational Studies in Epidemiology (STROBE) statement: guidelines for reporting observational studies. Lancet 370(9596):1453–1457. 10.1016/S0140-6736(07)61602-X. /10/20 2007 18064739

[20] About us | The EQUATOR Network

[21] Rubin DB (1996) Multiple Imputation after 18 + Years. Journal of the American Statistical Association. /06/01 1996;91(434):473–489. 10.1080/01621459.1996.10476908

[22] Schafer JL (1997) Analysis of Incomplete Multivariate Data

[23] VanderWeele TJ, Ding P (2017) Sensitivity Analysis in Observational Research: Introducing the E-Value. Ann Intern Med. /08/15 2017;167(4):268–274. 10.7326/M16-2607 28693043

[24] van de Schoot R, Depaoli S, King R (2021) Bayesian statistics and modelling. Nat Rev Methods Primers. /01/14 2021;1(1):1–26. 10.1038/s43586-020-00001-2

[25] Kruschke JK (2015) Highest Density Interval - an overview. Doing Bayesian Data Anal

[26] Gelman A, Rubin DB (1992) Inference from Iterative Simulation Using Multiple Sequences. Statistical Science. /11 1992;7(4):457–472. 10.1214/ss/1177011136

[27] Brooks SP, Gelman A (1998) General Methods for Monitoring Convergence of Iterative Simulations. Journal of Computational and Graphical Statistics. /12/01 1998;7(4):434–455. 10.1080/10618600.1998.10474787

[28] Stata. 17 ed. (2021)

[29] Thomas SV, Koshy S, Nair CR, Sarma SP (2005) Frequent seizures and polytherapy can impair quality of life in persons with epilepsy. Neurol India Mar 53(1):46–50. 10.4103/0028-3886.15054 15805655

[30] Lee JW, Dworetzky B (2010) Rational Polytherapy with Antiepileptic Drugs. Pharmaceuticals (Basel). Jul 26.;3(8):2362–2379. 10.3390/ph3082362 27713357 PMC4033928

[31] Conte V, Carrabba G, Magni L (2015) Risk of perioperative seizures in patients undergoing craniotomy with intraoperative brain mapping. Minerva Anestesiol 2015/04(4):379–388 25057931

[32] Ersoy TF, Ridwan S, Grote A, Coras R, Simon M (2020) Early postoperative seizures (EPS) in patients undergoing brain tumour surgery. Scientific Reports. /08/13 2020;10(1):13674. 10.1038/s41598-020-70754-z 32792594 PMC7426810

[33] Karakis I, Leeman-Markowski BA, Leveroni CL Intra-stimulation discharges: An overlooked cortical electrographic entity triggered by direct electrical stimulation. Clin Neurophysiol. 2015/05/01 2015;126(5):882–888. 10.1016/j.clinph.2014.08.011 25266305

[34] Cordella R, Acerbi F, Marras CE (2013) Risk of seizures during intraoperative electrocortical stimulation of brain motor areas: a retrospective study on 50 patients. Neurol Sci 2013/01(1):63–70. 10.1007/s10072-012-0968-2 22350148

[35] Kalamangalam GP, Tandon N, Slater JD (2014) Dynamic mechanisms underlying afterdischarge: a human subdural recording study. Clin Neurophysiol. /07 2014;125(7):1324–1338. 10.1016/j.clinph.2013.11.027 24365519 PMC6827874

[36] Pouratian N, Cannestra AF, Bookheimer SY, Martin NA, Toga AW (2004) Variability of intraoperative electrocortical stimulation mapping parameters across and within individuals. J Neurosurg. /09 2004;101(3):458–466. 10.3171/jns.2004.101.3.0458 15352604

[37] Lesser RP, Lee HW, Webber WRS, Prince B, Crone NE, Miglioretti DL (2008) Short-term variations in response distribution to cortical stimulation. Brain 2008/06(Pt 6):1528–1539. 10.1093/brain/awn044 18337272 PMC2408939

[38] Chaichana KL, Parker SL, Olivi A, Quiñones-Hinojosa A (2009) Long-term seizure outcomes in adult patients undergoing primary resection of malignant brain astrocytomas. Clinical article. J Neurosurg Aug 111(2):282–292. 10.3171/2009.2.Jns081132 19344222

[39] Chauhan P, Philip SE, Chauhan G, Mehra S (2022) The Anatomical Basis of Seizures. In: Czuczwar SJ, ed. Epilepsy. Exon Publications Copyright: The Authors.; The authors confirm that the materials included in this chapter do not violate copyright laws. Where relevant, appropriate permissions have been obtained from the original copyright holder(s), and all original sources have been appropriately acknowledged or referenced

[40] Suarez-Meade P, Marenco-Hillembrand L, Prevatt C (2020) Awake vs. asleep motor mapping for glioma resection: a systematic review and meta-analysis. Acta Neurochirurgica. /07/01 2020;162(7):1709–1720. 10.1007/s00701-020-04357-y 32388682

[41] Englot DJ, Han SJ, Berger MS, Barbaro NM, Chang EF (2012) Extent of Surgical Resection Predicts Seizure Freedom in Low-Grade Temporal Lobe Brain Tumors. Neurosurgery 70(4):921–928. 10.1227/NEU.0b013e31823c3a30 21997540

[42] Youngerman BE, Joiner EF, Wang X (2020) Patterns of seizure prophylaxis after oncologic neurosurgery. Journal of Neuro-Oncology. /01 2020;146(1):171–180. 10.1007/s11060-019-03362-1 31834582

[43] Wali AR, Rennert RC, Wang SG, Chen CC (2017) Prophylactic anticonvulsants in patients with primary glioblastoma. J Neurooncol 2017/11(2):229–235. 10.1007/s11060-017-2584-8 28755321

[44] Kruschke JK, Liddell TM (2018) Bayesian data analysis for newcomers. Psychon Bull Rev. /02 2018;25(1):155–177. 10.3758/s13423-017-1272-1 28405907

[45] Yuan Y, MacKinnon DP (2009) Bayesian mediation analysis. Psychol Methods. /12 2009;14(4):301–322. 10.1037/a0016972 19968395 PMC2885293

[46] Rendón-Macías ME, Riojas-Garza A, Contreras-Estrada D, Martínez-Ezquerro JD (2018) [Bayesian analysis. Basic and practical concepts for its interpretation and use]. Rev Alerg Mex 2018/09(3):285–298. 10.29262/ram.v65i3.512 30176207

